# Behavior Change Techniques and the Effects Associated With Digital Behavior Change Interventions in Sedentary Behavior in the Clinical Population: A Systematic Review

**DOI:** 10.3389/fdgth.2021.620383

**Published:** 2021-07-08

**Authors:** Jaime Martín-Martín, Cristina Roldán-Jiménez, Irene De-Torres, Antonio Muro-Culebras, Adrian Escriche-Escuder, Manuel Gonzalez-Sanchez, María Ruiz-Muñoz, Fermin Mayoral-Cleries, Attila Biró, Wen Tang, Borjanka Nikolova, Alfredo Salvatore, Antonio I. Cuesta-Vargas

**Affiliations:** ^1^Faculty of Health Sciences, Instituto de Investigación Biomédica de Málaga (IBIMA), Málaga, Spain; ^2^Grupo de Clinimetria (FE-14), Instituto de Investigación Biomédica de Málaga (IBIMA), Málaga, Spain; ^3^Faculty of Medicine, Department of Human Anatomy, Legal Medicine and History of Science, Legal Medicine Area, University of Malaga, Malaga, Spain; ^4^Physical Medicine and Rehabilitation Unit, Regional University Hospital of Malaga, Malaga, Spain; ^5^Instituto de Investigación Médica de Málaga, IBIMA, Malaga, Spain; ^6^Mental Health Unit, Regional University Hospital of Malaga, Malaga, Spain; ^7^ITWare, Budapest, Hungary; ^8^Faculty of Science and Technology, Bournemouth University, Poole, United Kingdom; ^9^Arthaus, Production Trade and Service Company Arthaus Doo Import-Export Skopje, Skopje, Macedonia; ^10^Sensor ID Snc, Boiano, Italy; ^11^School of Clinical Science, Faculty of Health Science, Queensland University Technology, Brisbane, QLD, Australia

**Keywords:** behavior, change, technique, digital intervention, sedentary behavior

## Abstract

**Background:** Sedentary behavior (SB) negatively impact health and is highly prevalent in the population. Digital behavior change interventions (DBCIs) have been developed to modify behaviors such as SB by technologies. However, it is unknown which behavior change techniques (BCTs) are most frequently employed in SB as well as the effect associated with DBCIs in this field. The aim of this systematic review was: (a) to evaluate the BCT most frequently employed in digital health including all technologies available and interventions aimed at increasing physical activity (PA), reducing sedentary time, and improving adherence to exercise in the clinical population, and (b) to review the effect associated with DBCIs in this field.

**Methods:** The database used was Medline, as well as Scopus, Scielo, and Google Scholar. For the search strategy, we considered versions of behavior/behavioral, mHealth/eHealth/telemedicine/serious game/gamification. The terms related to PA and SB were included, the criteria for inclusion were randomized clinical trials (RCTs), adults, intervention based on digital media, and outcome variable lifestyle modification; a last 5 years filter was included. Michie's Taxonomy was used to identify BCTs. The study was registered under the number PROSPERO CRD42019138681.

**Results:** Eighteen RCTs were included in the present systematic review, 5 of them healthy adults, and 13 of them with some illness. Studies included 2298 sedentary individuals who were followed up for 5 weeks−3 years. The most used BCTs were goal setting, problem solving, review outcomes/goals, feedback on behavior and outcomes of behavior, self-monitoring of behavior, social support, information about health consequences, and behavior practice/rehearsal. The effect associated with DBCIs showed improvements, among several related to PA and physiologic self-reported and anthropometric outcomes.

**Conclusion:** The BCTs most used in digital health to change outcomes related to SB were goals and planning, feedback and monitoring, social support, natural consequences, repetition, and substitution. Besides these findings, DBCIs are influenced by several factors like the type of intervention, patients' preferences and values, or the number of BCTs employed. More research is needed to determine with precision which DBCIs or BCTs are the most effective to reduce SB in the clinical population.

## Introduction

Our daily behavior heavily influences our health. According to the County Health Rankings, a 30% variation in health can be accounted for by healthy behaviors, such as smoking, alcohol intake, diet, and physical activity (PA) levels (1). Regarding PA, the World Health Organization (WHO) recommends a minimum of moderate and vigorous-intensity PA per week (2). The WHO defined moderate-intensity PA as any activity with a metabolic equivalent of task (MET) value between 3 and 5, and vigorous intensity PA as ≥6 MET. More recently, the term “sedentary behavior” (SB) was introduced to define any behavior characterized by energy expenditure of 1.5 MET or less, undertaken while in a sitting or reclining posture (3).

The latest research has shown that prolonged SB is positively associated with a range of health concerns including all-cause mortality, cardiovascular disease, type 2 diabetes, metabolic syndrome, and several types of cancers (4, 5). Despite this harmful effect to health, modern society provides many opportunities for prolonged sitting (6) and SB is highly prevalent in the majority of people's time (55–69% of the day) (7). Therefore, behavior change in SB is of great interest.

Behavior change techniques (BCTs) have been defined as irreducible, observable, and replicable components of an intervention designed to redirect behavior (8). In this way BCTs refer to a component of an intervention designed to alter or redirect causal processes that regulate behavior. In 2013, Michie et al. classified 93 hierarchically clustered techniques, distributed in 16 groups, called BCT Taxonomy (v1) (9). The BCT Taxonomy helps to identify the effectiveness of BCTs, including those developed by apps for physical activity (10, 11). According to Yardley et al. (12), digital behavior change interventions (DBCIs) employ digital technologies to support behavior change and can be used to promote health.

Given the rise of new technologies, DBCIs have been developed using technologies such as mobile applications and websites (13). Technology-enhanced solutions such as mobile applications, activity monitors, prompting software, texts, emails, and websites are being harnessed to reduce SB (14). Previous systematic reviews and meta-analyses have studied the beneficial effect of DBCIs on SB by both increasing PA or reducing sedentary time (14–16). However, one of these meta-analyses studied technologies limited to computer, mobile, and wearable technology devices in a healthy population (14); and another meta-analysis including all digital devices/technologies, focused on older adults aged ≥50 years (15).

Currently, there have been different systematic reviews that have studied the beneficial effect of DBCIs on SB by increasing PA, reducing sedentary time, and improving adherence to exercise for digital health in people with different clinical conditions. A systematic review with a meta-analysis (2017) aimed at evaluating the effectiveness of digital technology-enhanced interventions to reduce SB as well as to examine the BCTs used. However, this study was limited to computer, mobile, and wearable technology devices in a healthy population (14). Secondly, there was more recent study (2019) with the design of a systematic review that addressed a wider range of technology devices under the name of Human Computer Interaction. However, it targeted several behaviors. In addition to physical activity and sedentary behavior, it included diet, sleep, smoking, and others. Furthermore, this study also focused on the healthy population (16). In addition to that, another recent systematic review with meta-analysis (2019) was aimed at assessing the efficacy of DBCIs on PA and SB. Although this study included all digital technologies and included people with different clinical conditions, it only focused on older adults aged ≥50 years (15). However, the BCTs used in DBCIs and their usability to reduce SB are not known.

Would it be possible to reduce SB and modify PA habits through exercise proposals with DBCIs in clinical and healthy populations according to published research? The growing research of DBCIs aimed at decreasing SB and the general interest in people with different clinical conditions (also named clinical population) make it necessary to revise this topic. Therefore, this systematic review's objective was to identify more frequently used BCTs and explore the effectiveness of DBCIs used in health interventions to reduce SB, increase PA, and improve adherence to exercise in clinical and healthy populations.

## Methods

A systematic review was conducted following PRISMA 2009 guidelines (17). This study aimed to do a systematic review of the evidence concerning the most frequently used BCTs in digital health and the effect associated with DBCIs for reducing SB by increasing PA levels, reducing sedentary time, and improving adherence to exercise in adults. PROSPERO study registration number: CRD42019138681.

To be included in this review, studies had to meet the criteria outlined below (based on the PICO model).

Population: Adults (healthy or clinical population).

Intervention: Exercise proposals through DBCIs with the aim of reducing SB, increasing PA, or exercise adherence.

Control/comparison: Non-DBCIs or general clinical practice.

Outcomes: Exercise questionnaires, clinical variables, exercise measurements (e.g., time registry, inertial sensors).

The database used was Medline through the search engines Pubmed, CINAHL, PEDro, and EMBASE. Results were completed with the databases Scopus, Scielo, and Google Scholar. A filter of the last five years was applied to obtain the best possible evidence. The search was performed between May and June, 2019.

For the search strategy, we considered different variations of the terms (“behavior” OR “behavior” OR “behavioral”) AND (“mHealth” OR “eHealth” OR telemedicine OR “gamification” OR “serious game”). In the same way, the terms related to sedentary behavior (“sedentar^*^ behavior^*^),” levels of PA (“exercise^*^,” “physical activity”), and adherence (“adherence”) to exercise were included.

The inclusion criteria for the studies were randomized clinical trials, PEDro scale score more than 5 (18), adult population, intervention based on digital media (mobile phone, tablet, computer, web systems, and recommender systems), BCTs used, and outcome variable lifestyle modification (related to PA, SB, or exercise adherence). The languages of the manuscripts should be English, French, Italian, Portuguese, or Spanish. The defined exclusion criteria were systematic reviews, meta-analyses, no use of BCTs, and absence of goals related to SB or PA.

**Figure 1:**
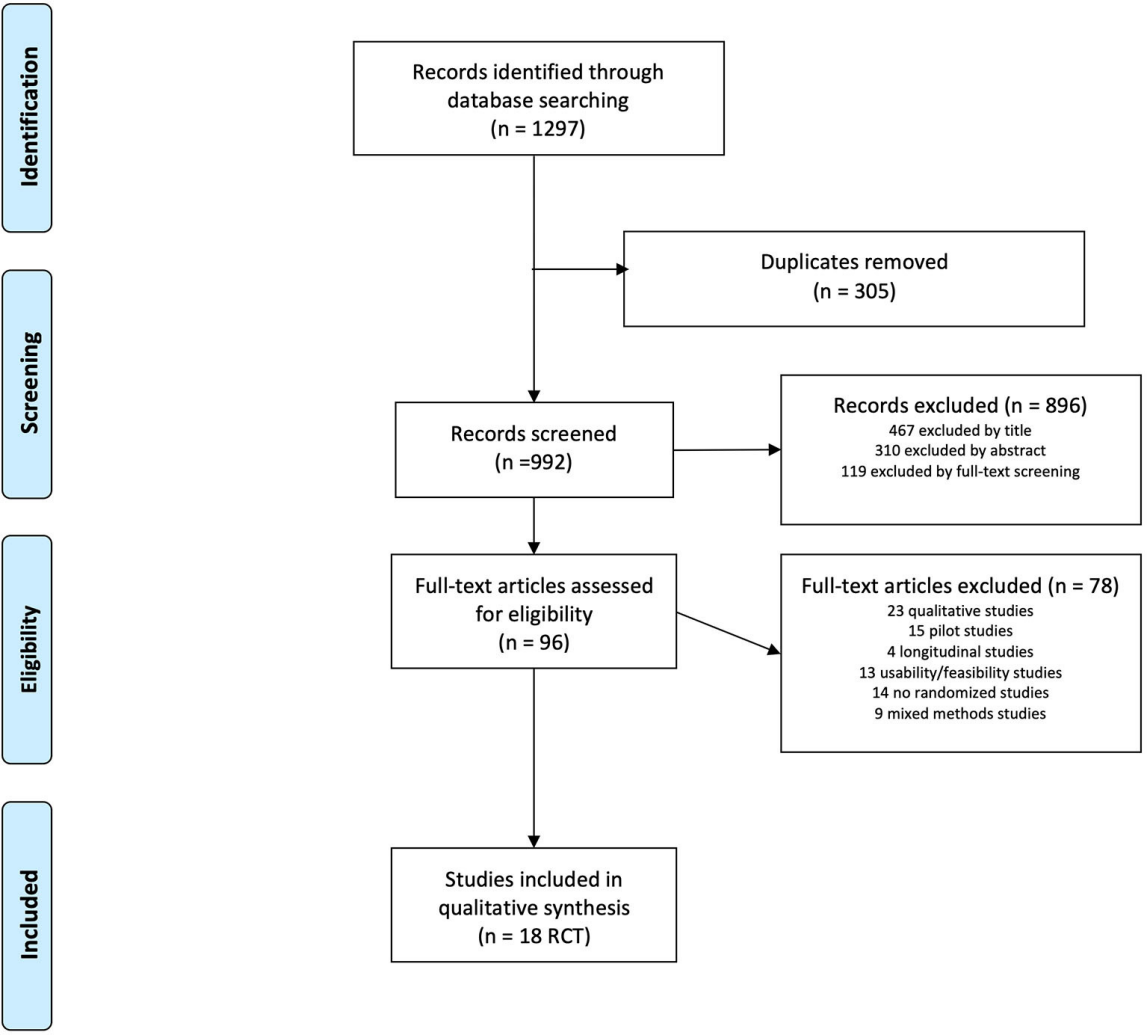
Selection study. Prisma 2009 flow diagram.

Three independent reviewers participated in each of the study selection phases. The search results were filtered by title and abstract according to eligibility criteria before full reading. Conflicts of inclusion were resolved by agreement between reviewers or by a fourth reviewer. A 4-phase flow chart of PRISMA (17) was performed to correctly stratify the results of the search (Figure 1). The internal and external validity of the manuscript included in the systematic review was evaluated by means of the PEDro scale (18) (Table 1). For each of the manuscripts included in the review, the following elements were identified: author, year of publication, population, age of the groups involved, distribution of the groups (Table 2), outcome related to SB, PA, or exercise adherence, and change relative to BCT (Table 3). We used Michie's Taxonomy to identify the different BCTs used in the studies (9).

**Table 1:** Validity of studies based on the PEDro scale.

	**Eligibility** ** criteria**	**Random** ** allocation**	**Concealed** ** allocation**	**Baseline** ** comparability**	**Blind** ** subjects**	**Blind** ** therapists**	**Blind** ** assessors**	**Adequate** ** follow-up**	**Intention-** **to-treat** ** analysis**	**Between-group** ** comparisons**	**Point estimates and variability**	**Total**
Adams et al. (19)	√	√	0	√	0	0	√	√	√	√	√	8/11
Frederix (20)	√	√	0	√	0	0	√	√	√	√	√	8/11
Haggerty et al. (21)	√	√	0	√	0	0	√	0	0	√	0	5/11
Haller et al. (22)	√	√	√	√	0	0	0	0	√	√	√	7/11
Holmen et al. (23)	√	√	√	√	0	0	√	0	√	√	0	7/11
Hutchesson et al. (24)	√	√	√	√	√	√	√	√	√	√	√	11/11
Kempf et al. (25)	√	√	√	√	0	0	0	√	0	√	0	6/11
Lari et al. (26)	√	√	0	√	0	0	0	√	√	√	√	7/11
Li et al. (27)	√	√	0	√	0	0	0	√	√	√	√	7/11
Maddison et al. (28)	√	√	0	√	0	0	0	√	√	√	√	7/11
Martin et al. (29)	√	√	√	√	0	0	0	√	√	√	√	8/11
Partridge et al. (30)	√	√	√	√	0	0	0	√	0	√	0	6/11
Pfaeffli Dale et al. (31)	√	√	0	0	0	0	0	0	√	√	√	5/11
Sharma et al. (32)	√	√	0	√	0	0	0	√	0	√	0	5/11
Short et al. (33)	√	√	√	√	0	0	0	0	√	√	√	7/11
Simons et al. (34)	√	√	0	√	0	0	0	√	0	√	0	5/11
Spring et al. (35)	√	√	√	√	0	0	√	0	0	√	√	7/11
Wayne et al. (36)	√	√	√	√	0	0	0	0	√	√	√	7/11

**Table 2:** Population data of the 18 randomized control trials that report behavior change techniques used in digital health interventions for reducing sedentary behavior.

**References**	**Population and sample size (*N*)**	**Follow-up time frame**	**Intervention group *N* (female percentage when reported)**	**Control group *N* (female percentage when reported)**	**Age (mean** **±** **SD, when reported)**		
					**Intervention group**	**Control group**	**Total**
Adams et al. (19)	Overweight/obese *N* = 96	4 months	G1 immediate rewards 25 (88% female) G2 delayed rewards 24 (71% female) G3 static goals with immediate rewards 24 (79% female) G4 static goals with delayed rewards 23 (70% female)		G1 41·0 ± 10·16; G2 44·5 ± 10·70; G3 38·4 ± 8·22; G4 40·3 ± 7·91		41 ± 9·5
Frederix (20)	Heart disease *N* = 126	6 months	62 (16% female)	64 (20% female)	61 ± 9	61 ± 8·0	
Haggerty et al. (21)	Endometrial cancer *N* = 19	6 months	11 (100% female)	8 (100% female)			62 ± 58·7
Haller (22)	Depression *N* = 20	4 months	14 (71% female)	6 (50% female)	43 ± 14·0	51 ± 12·0	45 ± 14·0
Holmen (23)	Type 2 diabetes *N* = 101	1 year	51(33% female)	50 (40% female)	58·6 ± 11·8	56 ± 12·2	
Hutchesson et al. (24)	Young women *N* = 57	6 months	29 (100% female)	28 (100% female)	26·3 ± 4·3	28 ± 5·0	27 ± 4·7
Kempf et al. (25)	Overweight *N* = 100	6 months	34 (15% female)	Control 1: 30 (16% female) Control 2: 36 (17% female)	51 ± 6	Control 1: 48 ± 5;	
						Control 2: 51 ± 5	
Lari et al. (26)	Type 2 diabetes *N* = 34	4 months	17 (46% female)	17 (47% female)	46 ± 9·1	49 ± 9·1	
Li et al. (27)	Knee osteoarthritis *N* = 61	6 months	61 (82% female)		62 ± 8·9		
Maddison et al. (28)	Heart disease *N* = 162	6 months	82 (16% female)	80 (13% female)	61 ± 13·2	62 ± 12·2	
Martin et al. (29)	Heart disease *N* = 48	3 weeks	G1 no texts 16 (44% female); G2 texts 16 (50%female)	16 (44% female)	G1 58 ± 8; G2 55 ± 8	60 ± 7·0	58 ± 8·0
Partridge et al. (30)	Healthy *N* = 248	9 months	123 (61% female)	125 (61% female)			
Pfaeffli Dale et al. (31)	Heart disease *N* = 123	6 months	61 (21% female)	62 (16% female)	59 ± 10·5	60 ± 11·8	59 ± 11·1
Sharma et al. (32)	Healthy *N* = 382	1 year	190 (51% female)	192 (53% female)	18–24 (21.1%); 25–34 (24.7%); 35–44 (22.1%); 45-54 (27.4%); 55–64 (4.7%)	18–24 (21.4%); 25–34 (24%); 35–44	
						(25.5%); 45–54 (26%); 55–64 (3.1%)	
Short et al. (33)	Breast cancer *N* = 156	3 months	156 (100% female)		55 ± 9·73		
Simons et al. (34)	Young adults *N* = 130	21 weeks	60 (42% female)	70 (60% female)	24·8 ± 3·1	25 ± 3·0	25
Spring et al. (35)	Healthy *N* = 128	9 months	84 (76% female)	44 (75% female)	41 ± 11·9	41 ± 10·9	41 ± 11·9
Wayne et al. (36)	Type 2 diabetes *N* = 97	6 months	48 (65% female)	49 (80% female)	53 ± 10·9	53 ± 11·9	

**Table 3:** Behavior change technique used in the selected studies.

		**Total *N* = 18 (100%)**	**References**
Goals and planning	Goal setting (behavior)	10 (56%)	(19, 27, 28, 30, 31, 33–36)
	Problem solving	5 (28%)	(24, 26, 32–34)
	Action planning	4 (22%)	(19, 25, 27, 33)
	Review behavior goal(s)	2 (11%)	(25, 30)
	Review outcome goal(s)	5 (28%)	(22, 25, 28, 29, 34)
Feedback and monitoring	Monitoring of behavior by others without feedback	2 (11%)	(23, 26)
	Feedback on behavior	6 (33%)	(20, 23, 25, 27, 29, 32)
	Self-monitoring of behavior	9 (50%)	(17, 19, 19, 23, 23, 24, 24, 27, 28, 28–30, 32, 33, 33–36)
	Self-monitoring of outcome(s) of behavior	2 (11%)	(31, 33)
	Biofeedback	2 (11%)	(23, 28)
	Feedback on outcome(s) of behavior	6 (33%)	(21, 22, 25, 29, 33, 35)
Social support	Social support (unspecified)	5 (28%)	(21, 24, 26–28)
	Social support (practical)	3 (17%)	(31, 33, 34)
Natural consequences	Information about health consequences	6 (33%)	(26, 29, 30, 32–34)
	Salience of consequences	1 (6%)	(32)
Comparison of behavior	Demonstration of the behavior	1 (6%)	(27)
	Social comparison	1 (6%)	(27)
Repetition and substitution	Behavioral practice/rehearsal	6 (33%)	(19, 21, 23, 24, 29, 33)
	Behavior substitution	1 (6%)	(24)
	Habit formation	4 (22%)	(21, 24, 28, 32)
Reward and threat	Social reward	1 (6%)	(29)
	Incentive (outcome)	2 (11%)	(19, 31)
	Reward (outcome)	1 (6%)	(27)
Scheduled consequences	Reduce reward frequency	1 (6%)	(30)

## Results

We identified 1297 articles in five searches. We retrieved 96 papers for full-text review, of which 18 RCT studies were identified as eligible for inclusion (Figure 1). Therefore, we analyzed individual data from the 18 RCTs with the PEDro internal validity scale (Table 1).

They all provided data on BCTs used in DBCIs to change SB. Studies population were all sedentary subjects, 5 of them healthy adults (24, 30, 32, 34, 35), and 13 of them with some pathological diagnosis [overweight/obese (19, 25), type 2 diabetes mellitus (23, 26, 36), heart disease (20, 28, 29, 31), cancer (21, 33), depression (22), and osteoarthritis (27) (Table 2)]. These studies included 2,298 individuals who were followed up for 5 weeks−3 years.

BCTs from Michie's Taxonomy were identified in the 18 RCTs. The most used were goal setting, problem solving, review outcomes/goals, feedback on behavior and outcomes of behavior, self-monitoring of behavior, social support, information about health consequences, and behavior practice/rehearsal (Table 3). All the interventions used text messages and most of them added other communication tools (calls, emails, among others), mobile apps, and some type of activity tracker. Types of interventions, control comparison, and outcomes measured in the 18 RCTs can be seen in Table 4, where the reader may also find the effects of intervention associated with DBCIs.

**Table 4:** Outcomes of interest of the selected studies.

**Author**	**Intervention**	**Control group**	**Outcome measurements**	**Data collection**	**Effects of intervention associated with DBCIs**
Adams et al. (19)	-Walking intervention through texting (WalkIT) focused on goal setting (adaptive vs. static goals) -Financial rewards (immediate rewards for PA goal attainment vs. delayed rewards for study participation).	-G1: immediate rewards. -G2: delayed rewards. -G3: static goals with immediate rewards. -G4: static goals with delayed rewards.	-Pedometer-measured steps per day. -Moderate vigorous physical activity minutes per day.	-Participants reported by survey their socio-demographic data. -BMI (kg/m2) was determined from researcher-measured height and weight.	-The static goal group trajectory crossed and dropped below the activity of the adaptive groups within four months, even with immediate rewards. -The implication is that adaptive goals will not result in immediate changes as large as static goals, but can contribute to a more gradual and perhaps more sustainable behavior change process over four months.
Frederix et al. (20)	An Internet-based, comprehensive, and patient-tailored telerehabilitation program with short message service (SMS) texting support for cardiac patients.	Conventional cardiac rehabilitation alone.	-International Physical Activity Questionnaire (IPAQ). -Health-related quality of life (HRQoL). -VO2 peak. -Accelerometry daily step counts.	-Two independent investigators, blinded to treatment allocation, interpreted cardiopulmonary exercise test reports. -The first secondary outcome measure was daily physical activity, both registered by triaxial accelerometry (Yorbody sensor) and self-assessed by the patient. -Qualitative feedback on the cardiac telerehabilitation system was obtained from intervention group patients by special offline feedback forms	-Control group patients (only presential cardiac rehabilitation program) return to prior lifestyle behavior after center-based cardiac rehabilitation, whereas intervention group patients (presential + tele-rehabilitation program) further maintain and ameliorate acquired behavioral change, thanks to adaptive goals, self-monitoring, and feedback.
Haggerty et al. (21)	Telemedicine with text messaging (texting).	Enhanced usual care.	-Weight change (kg). -% total weight loss. -Waist circumference change (cm). -Physical health component (SF-12). -Cancer-related body image. -Female sexual functioning—satisfaction. -Walking activity (METs/wk; IPAQ). -Total Physical Activity (METs/wk; IPAQ). -Vigorous Physical Activity (METs/wk; IPAQ).	-Body weight was measured using a calibrated digital scale. -Height was measured using a stadiometer. -Waist circumference was measured according to the WHO Physical Measurements Guidelines. -Participants completed several validated psychosocial assessment instruments.	-Weight loss and improved quality of life through texting, goals, and natural consequences.
Haller et al. (22)	-Access to a home page and a heart rate monitor (Polar FT1; Polar Electro, Büttelborn, Germany) and four different types of resistance bands (Thera-Band, Akron, OH, United States). -Message function was used to send exercise schedules to the patients once weekly. -After each week, motivational feedback was given to improve adherence. -At the end of each week, patients were expected to upload a protocol of their weekly activity on the platform, making the protocol available to the supervisor. -Based on this response, training goals were individually adapted in the duration and intensity for the following week to keep motivation high and prevent patients from overload and frustration.	Treatment-as-usual.	-Adherence. -Depression Scales (clinician rating, QIDS-C, and self-report, QIDS-SR). -The Short Form-36 (SF-36). -Quality of Life questionnaire. -Treadmill walking test until exhaustion to determine peak oxygen uptake (VO2 peak). -Lactate threshold.	After clinical rating and answering questionnaires, all patients performed a treadmill walking test until exhaustion to determine peak oxygen uptake (VO2 peak) and lactate threshold.	Web-based exercise intervention using goal-planning led to a significant and clinically relevant improvement of depressive symptoms.
Holmen et al. (23)	-Mobile phone–based self-management system Few Touch Application (FTA). -The FTA consisted of a blood glucose–measuring system with automatic wireless data transfer, diet manual, physical activity registration, and management of personal goals, all recorded and operated using a diabetes diary app on the mobile phone. -In addition, health counseling based on behavior change theory and delivered by a diabetes specialist nurse for the first four months after randomization.	-Mobile phone–based self-management system used for one year. -With or without telephone health counseling by a diabetes specialist nurse for the first four months.	-HbA1c level. -Self-management (heiQ). -Health-related quality of life (SF-36). -Depressive symptoms (CES-D). -Lifestyle changes (dietary habits and physical activity).	-Demographic information were self-reported. -Clinical data were obtained from the General Practitioners or self-reported.	-They used personalized goals, self-monitoring, and natural consequences to obtain improvement in HbA1c level. -Self-management (heiQ), health-related quality of life (SF-36), depressive symptoms (CES-D), and lifestyle changes (dietary habits and physical activity).
Hutchesson et al. (24)	Intervention Be Positive Be Health*e* (BPBH): a six-month weight loss program delivered using e-Health technologies only, comprising five delivery modes (website, app, email, text messages, and social media) and using social cognitive theory and control theory theoretical frameworks.	Waiting list.	-Weekday sitting time (minutes/day). -Weekend sitting time (minutes/day). -Total sitting time (minutes/day). -Energy intake (kJ/day). -Fruit (% energy/day). -Fruit (grams/day). -Vegetable (% energy/day). -Vegetable (grams/day). -Alcohol (% energy/day). -Alcohol (grams/day). -Takeaway (% energy/day). -% energy from non-core foods. -% energy from core foods. -QLESQ total score. -Satisfaction with life scale.	-Height, weight, waist circumference, and blood pressure measurements were taken twice for accuracy, with a third measurement also taken in cases where either of the first two values fell outside a predetermined acceptable range. -An online survey assessed physical activity, sitting time, dietary intake, and quality of life.	-They found useful social support to obtain positive improvements in body fat and dietary intake.
Kempf et al. (25)	Telemedical coaching program accompanied with telemonitoring (scales, pedometers, coaching with weekly care calls).	-Telemedical -Coaching program without telemonitoring.	-Difference in body weight reduction -Freiburger Questionnaire for Physical Activity (FFkA). -Sport per week hour; physically active per week hour.	Self-reported.	Self-monitoring and feedback were useful in weight reduction.
Lari 2018 (26)	Training messages within two weeks (two or three messages daily) in the field of physical activity based on health promotion model constructs.	No intervention.	-V°O2 max at 12 weeks. -Weight. -Body mass index. -Waist/hip circumference. -Physical activity (accelerometry). -Exercise-related motivation. -Exercise adherence. -Health-related quality of life (HRQoL).	-The researchers administered the physical activity questionnaire through interviews and monitored the questionnaires completion. -The heights and weights of the participants were measured to determine the METs and BMIs.	-Training messages were useful in changing physical activity behaviors of diabetic patients and their beliefs: perceived self-efficacy, family support and lower perceived barriers.
Li et al. (27)	-Brief education session by a physical therapist. -Fitbit Flex. -Four biweekly phone calls for activity counseling.	Waiting list.	-MET. -3-part-questionnaire: perceived health status, HPM Constructs (barriers, social support…) and Physical Activity 7-day.	-Self reported. -For sedentary activities, they calculated the mean daily time spent with an energy expenditure of ≤ 1.5 METs, occurring in bouts of 20 minutes or more during waking hours.	-A counseling program with tools as natural consequences, self-monitoring, feedback and goal-planning, improved METs, daily steps, activity of daily living, and quality of life.
Maddison et al. (28)	-12 weeks of telerehabilitation. -REMOTE-CR provided individualized exercise prescription. -Real-time exercise monitoring/coaching and theory-based behavioral strategies via a bespoke -Telerehabilitation platform.	Center-based rehabilitation.	-Change in Steps/Day (accelerometry). - Total and Aerobic Activity Times.	-Automatically captured. -Self reported. -Hospital service and medication utilization were extracted from the New Zealand Ministry of Health National Minimum Dataset and Pharmaceuticals Collection.	-They used self-monitoring, feedback, goal setting, and social support. Participants were less sedentary at 24 weeks. Participants had smaller waist and hip circumferences at 12 weeks.
Martin et al. (29)	-Participants used their own smartphones. -Digital physical activity tracking was performed using the Fitbug Orb (Chicago, IL). -They used sequential randomization to individually evaluate the tracking and texting components of the intervention. -After establishing baseline activity during a blinded run-in (week 1), in phase I (weeks 2 to 3) they randomized 2:1 to unblinded versus blinded tracking. -The activity tracker itself did not show activity information, but continuously transmitted it in all participants. -In phase II (weeks 4 to 5), they randomized unblinded participants 1:1 to “smart texts” versus no texts. -Smart texts were automated, personalized, smartphone-delivered coaching messages informed by real-time activity.	-Blinded tracking. -No texts.	-IPAQ-Sf. -MET. -Physical Activity self-efficacy: mins/week; days/week.	-Automatically captured. -Self reported. -Participant satisfaction through an online survey.	-They used feedback and encouraging messages. -An automated tracking-texting intervention increased physical activity with, but not without, the texting component.
Partridge (30)	-mHealth-program, TXT2BFiT, consisting on personalized coaching calls with a dietitian based on motivational interviewing with goal setting and review. -Eight weekly text messages, personalized by gender and stage-of-change. -Weekly reminder emails. -Access to four behavior specific educational and self-monitoring smartphone applications and the study website for education.	-Two-page handout based on the Australian Dietary Guidelines and National Physical Activity Guidelines considered as usual care. -An introductory phone call (no coaching given). -Four text messages (one for each behavior) over the first three months.	-Body mass index. -Waist-to-hip ratio. -Blood pressure. -Cholesterol. -CVD risk probability. -Medication adherence. -Overall illness threat. -Hospital Anxiety and Depression Scale (HADS). -Overall self-efficacy.	-Self-reported. -Collected by clinical practitioners.	-The intervention used natural consequences, self-monitoring, and messages with effect on physical activity (MET minutes per week and physical activity days per week) at 3 months.
Pfaeffli Dale et al. (31)	-Personalized 24-week mHealth program. -Framed in social cognitive theory. -Sent by fully automated daily short message service (SMS) text messages. -A supporting website.	Usual care control.	-Patient adherence. -MET-min per week.	-Self-reported. -Collected by clinical practitioners.	-A mHealth intervention (messages and natural consequences) plus usual care showed a positive effect on adherence to multiple lifestyle behavior changes at three months. -Adherence to healthy lifestyle behaviors measured using a self-reported composite health behavior score.
Sharma et al. (32)	-Telephone call once a month. -The intervention package also included weekly text messages via SMS. -The text messages included short but catchy slogans and rhymes on the importance of modification of behavioral risk factors.	No mobile-health intervention.	-Moderate to vigorous aerobic activity min/week. -Resistance-training score (n° sessions × n° exercises).	-Self-reported. -Collected by clinical practitioners.	-After Intervention Phase (messages and calls), significant reduction was seen in behavioral risk factors (unhealthy diet and insufficient physical activity) in the intervention group compared to control group. -Body mass index (BMI), systolic blood pressure, and fasting blood sugar level also showed significant difference in the intervention group as compared to controls.
Short et al. (33)	-Monthly three-module intervention: -Module 1 comprised information on physical activity guidelines for cancer survivors. -Module 2 included feedback on physical activity behavior. -Module 3 included tailored feedback on physical activity performance.	-Single module group received the same psycho-social module content as participants in the three-module groups. -All content was delivered in a single module at week 1.	-Minutes of moderate to vigorous physical activity per day. -Sedentary leisure minutes per day. -Accelerometry. -Fruits and vegetables servings per day. -Saturated fat intake.	-Self-reported. -Collected by clinical practitioners. -Automatically captured.	-After goal setting and planning intervention, completers allocated rated higher on acceptability and higher levels of resistance-training.
Simons et al. (34)	-Active Coach app (for 9 weeks) combined with a Fitbit activity tracker. -Personal goals, practical tips, and educational facts were provided to encourage physical activity.	Print-based generic physical activity information.	-Change in HbA1c. -Weight. -Waist circumference. -Body mass index. -Satisfaction with life. -Hospital Anxiety and Depression Scale (HADS). -Positive and Negative Affect Schedule (PANAS). -Quality of life (Short Form Health Survey-12 [SF-12]).	-Self-reported. -Collected by clinical practitioners. -Automatically captured.	-No significant intervention effects were found for objectively measured physical activity, self-reported physical activity, and self-reported psychosocial variables. -The lack of significant intervention effects might be due to low continuous user engagement. -Advice or feedback was not perceived as adequately tailored.
Spring et al. (35)	-Smartphone app and accelerometer to track targeted behaviors and received personalized remote coaching from trained paraprofessionals. -Perfect behavioral adherence was rewarded with an incentive of US $5 per week for 12 weeks. -Moderate to vigorous physical activity either simultaneously or sequentially after other diet and activity risk behaviors information.	Stress and sleep contact.	-Diet. -Activity behaviors.	Self-reported.	-The unusually large, well-maintained diet and activity improvements observed in this trial are likely attributable to the effectiveness of the MBC2 intervention components, including the use of appealing mHealth technology and connective coaching as vehicles to deploy effective behavior change techniques (goal-setting, self-monitoring, feedback, support, accountability).
Wayne et al. (36)	-Six months of health coaching with mobile phone monitoring support. -The intervention group was provided with a Samsung Galaxy, they were also provided a user account with the Connected Wellness Platform (CWP), which supported participants in health-related goal setting and progress monitoring. -Participants could track key metrics, notably blood glucose levels, exercise frequency/duration/intensity, food intake, and mood. -They could communicate with their health coach at any time in the 24-hour cycle via secure messaging, scheduled phone contact, and/or during in-person meetings.	-Six months of health coaching without mobile phone monitoring support. -Exercise Education Program. -In-person meetings. -Health coach phone contacts.	-Change in HbA1c. -Weight. -Waist circumference. -Body mass index (BMI). -Satisfaction with life. -Depression and anxiety (Hospital Anxiety and Depression Scale [HADS]). -Positive and negative affect (Positive and Negative Affect Schedule [PANAS]). -Quality of life (Short Form Health Survey-12 [SF-12]).	-Self-reported. -Collected by clinical practitioners.	-The Connected Wellness Platform enabled self-monitoring and health coach interactions with intervention participants, providing a cloud-based platform for mobile phone-based health management. -This system provided secure, two-way communication between client and health coach, with mobile phone data entry on relevant behaviors entered manually.

## Discussion

This systematic review analyzed the BCTs most frequently used and the effectiveness of DBCIs used in health interventions aimed at reducing SB, increasing PA, and improving adherence to exercise in clinical and healthy populations.

Regarding the BCTs most frequently used in DBCIs to change outcomes related to SB, results showed that they were goals and planning, feedback and monitoring, social support, natural consequences, repetition, and substitution (Table 3). Therefore, we recognize them as the most important psychological strategies to improve SB change from the 19 groups of BCTs proposed by Michie et al. (9). These findings concur with previous literature showing that the most widely used BCTs are self-monitoring, goal-setting, credible source, feedback on behavior, and social support (13, 15). Among these groups of BCTs, the most frequently used was goal setting (*n* = 10) as part of the goals and planning cluster (Table 3). More specifically, it was used in five studies involving healthy adults aimed at promoting PA (19, 29, 30, 34, 35) and other five studies involving patients suffering from cardiac-related disease (28, 31), cancer (33), diabetes (36), or musculoskeletal impairments. (27). The second most used BCT was self-monitoring of behavior (*n* = 9) as part of the feedback and monitoring cluster. However, only two out of nine studies included healthy adults (19, 35). In light of these findings, goal setting is more frequently used in healthy populations than self-monitoring of behavior.

A previous meta-analysis limited to studies in a healthy population found that the most frequently used BCTs were “prompts and cues,”, “self-monitoring of behavior,” “social support,” and “goal settings,” which were coded between 10 and 5 times (14). However, in this study, only 20/93 available BCTs were used (14). Another meta-analysis that focused on adults aged ≥50 years found that the most common BCTs were “social support,” “goal setting,” “feedback on behavior,” and “self-monitoring” (15). These last three BCTs along with “credible source” comprise the most common BCTs in a meta-analysis targeting PA and diet in cancer survivors, although no studies were found to assess SB in this cancer population (16). These BCTs are among the most used in the present systematic review (Table 3).

Regarding the effect associated with DBCIs, the interventions included in the 18 RCTs showed improvements among several outcomes such as an increase in daily steps, MET, and International Physical Activity Questionnaire (IPAQ). When the clinical population (non-healthy) was evaluated, other complementary outcomes were addressed as well as improvements in self-reported outcomes such as quality of life, self-efficacy, or barriers to exercise, and improvements in anthropometric outcomes such as weight loss or body circumferences (Table 4). Smart texts were grouped as positive reinforcement messages, sent when a participant was on track to attain or had already attained his or her daily goal, and booster messages, to motivate individuals when they were not tracking to surpass their step goal (29, 32). Short message service (SMS) based on a health promotion model could be used to change beliefs (self-efficacy, family support, coping with barriers) and physical activity (25).

There is evidence that DBCIs to promote PA and/or reduce SB result in increases in total PA and reductions in SB in older adults aged ≥50 years, at least in the short term (15). Stephenson et al. (14), in their meta-analysis on interventions in the workplace, found that SB showed a mean reduction of 40 min/workday in the intervention group at the endpoint follow-up. Greater reductions in SB were found in studies where self-report/proxy measures (53 min/day) of SB were used compared to objective measures (35 min/day) (14).

Besides findings regarding the effects associated with DBCIs, the interventions employed among studies are not common, so it is not possible to know the implication of BCTs among interventions, which concur with previous literature. A systematic review showed that DBCIs are an effective tool to increase PA and reduce body mass index in cancer survivors. However, authors agreed that outcome measures were not common (13). Likewise, the theoretical models applied are not clearly described by the authors (13–15). In this way, risk of bias and heterogeneity of this type of study was high. This is due to the fact that there is a wide variability of DBCIs, different types of intervention, and different assessment instruments (13, 14). Despite this, studies show an increase in vigorous PA, a reduction in body mass index, and a reduction in fatigue in cancer patients (13). Also, the use of computer, mobile, and wearable technology have been shown to be effective tools to reduce SB. Likewise, the permanence of life habits acquired in different studies must be analyzed in the long term through follow-up and without complementary behavior change systems (15).

Besides differences in DBCIs employed, there are several factors that may influence their effectiveness. One of them is the time slot. For example, immediate rewards have been shown to be more effective at stimulating change behavior than delayed rewards in overweight/obese adults. In addition, adaptive goal-setting encourages better goal keeping in the long-term than static goals (19). Another factor is the number of BCTs employed. Joining different BCTs and self-monitoring of the behavior has a summative effect, as was seen in Kempf's study (25). Overweight adults equipped with telemonitoring devices (scales and pedometers) and supported by mental coaches, demonstrated large reductions of body weight after 12 months of lifestyle intervention and the within-group analysis revealed a larger reduction in body weight for the intervention with the combination of telemedical coaching and telemonitoring of PA than only telemonitoring (23). Wayne (36) shows similar data about the summative effect between coaching and monitoring, they found accelerated improvement in the group with mobile phone monitoring added to a health coaching strategy than in the group with coaching alone within a diabetes population. They describe a faster improved adoption and adherence to health behaviors with the combined strategy (36). In this way, delivering tailored modules on a monthly schedule and combining them with weekly action planning is a good example to improve PA in breast cancer survivors (33). The use of self-management techniques in exercise programs could enhance PA adherence among healthy subjects and the clinical population [e.g., knee osteoarthritis (27) or heart disease (29)], encouraging users to keep their motivation through the combination of message strategies and interventions similar to pedometer data through a Smartphone interface.

Lately, other factors influencing the effects of DBCIs have been difficult to analyze, like patients' preferences and values, namely the patient-centered approach. It is associated with a higher rate of patient satisfaction, better adherence to suggested lifestyle changes, and more cost-effective care (37). However, in most systematic reviews, a person-based approach is limited to a few examples, and there is a lack of defining user characteristics in digital health (12). Another factor is the number of BCTs employed, as evidence suggests that incorporating a multitude of BCTs is more effective than those using few or a single BCT in terms of long-term changes (38). For example, in older patients, a minimum of three groups of BCTs are necessary to obtain significant effects on PA (15). Finally, the social, cultural context and environment are key factors influencing SB and must be taken into consideration when developing DBCIs targeting SB (39).

However, the effectiveness and costs of real-time remote exercise monitoring and coaching to traditional face-to-face rehabilitation must be considered (28). This kind of intervention is at least as favorable as a face-to-face program. Immediate post intervention feedback and at longer-term follow-up (24 weeks) could be considered in this type of intervention (28). Moreover, remote intervention realized a 70% reduction in programmer delivery costs, and very few adverse events were attributed to participation in the programmer. Specialists provided real-time individualized audio coaching, feedback, and social support throughout real-time exercise monitoring (28).

The limitation of the present study refers to data analysis. This systematic review focused on the last five years due to the existence of similar studies; however, this ensured that the most recent studies would be captured. The studies included in the systematic review did not have an outcome variable measured with a common instrument and sometimes did not offer numerical data on the changes produced. This fact makes statistical treatment difficult through meta-analysis. For future RCTs evaluating DBCIs in SB that could be assessed in a meta-analysis, standardized outcomes would be recommended, at least the most studied, such as daily steps, MET, and levels of PA measured by IPAQ (see Table 4). In addition, current evidence showed a high heterogeneity in the clinical population. A greater number of RCTs would allow further meta-analysis in a given clinical population. Besides the mentioned limitations, this is the first study to analyze in depth and compare the results of applying BCTs in increasing PA or interrupting SB in different populations. The demonstrated effectiveness of BCTs in intervention protocols related to PA could be implemented in other types of treatment to increase adherence.

## Conclusion

The present systematic review showed that the most common BCTs aimed at reducing SB in DBCIs were goal setting, problem solving, review outcomes/goals, feedback on behavior and outcomes of behavior, self-monitoring of behavior, social support, information about health consequences, and behavior practice/rehearsal. The DBCIs among studies included revealed beneficials effects in outcomes such as an increase in MET, PA levels, or daily steps; improvements in self-reported outcomes such as quality of life, self-efficacy, or barriers to exercise; and improvements in anthropometric outcomes such as weight loss or body circumferences. Besides these findings, DBCIs are influenced by several factors like the type of intervention, patients' preferences, and values or the number of BCTs employed. Therefore, more research is required to determine with precision which DBCIs are the most effective to reduce SB in the clinical population.

## Data Availability Statement

The original contributions presented in the study are included in the article/Supplementary Material, further inquiries can be directed to the corresponding author/s.

## Author Contributions

JM-M, CR-J, and ID-T: data search. AC-V and WT: conceptualization. AE-E, AM-C, MR-M, and MG-S: formal analysis. AB, WT, AS, AC-V, BN, and FM-C: funding acquisition, project administration, and supervision. JM-M, CR-J, ID-T, and AC-V: methodology. JM-M, CR-J, ID-T, and MR-M: writing—original draft. JM-M, CR-J, I-DT, AE-E, AM-C, MR-M, MG-S, AB, WT, AS, AC-V, BN, and FM-C: writing—review and editing. All authors contributed to the article and approved the submitted version.

## Conflict of Interest

The authors declare that the research was conducted in the absence of any commercial or financial relationships that could be construed as a potential conflict of interest.
